# Identification and Behavioral Evaluation of Ketone Attractants for the Stable Fly, *Stomoxys calcitrans*

**DOI:** 10.3390/insects17101071

**Published:** 2026-10-16

**Authors:** Saravan Kumar Parepely, Abhinav Kumar Maurya, Dangsheng Liang

**Affiliations:** Apex Bait Technologies, Inc., Santa Clara, CA 95054, USA

**Keywords:** stable fly, *Stomoxys calcitrans*, attractant, olfactory lure, semiochemicals, ketones

## Abstract

The stable fly, *Stomoxys calcitrans* L., is one of the most damaging blood-feeding pests of livestock. Stable flies use odors associated with vertebrate hosts and their environments to locate blood meal and suitable oviposition and larval-development sites. Previous studies have identified behaviorally active gases, phenolic compounds, short-chain fatty acids, alcohols, and sulfur-containing compounds from cattle-associated odor sources, but ketones have received limited behavioral investigation. We first evaluated stable-fly attraction to host- and resource-associated substrates, including chicken manure, wet blood meal and sheep blood and characterized volatile compounds from attractive materials using SPME-GC-MS. We then tested ketones detected in the headspace of these substrates or reported in published studies. Three ketones, 1-octen-3-one, 3-octanone, and 6-methyl-5-hepten-2-one, significantly attracted adult stable flies. Following blend evaluation, 3-octanone and 1-octen-3-one were selected as the two-component K-lure blend which was significantly more attractive than the control in choice assays. Combining K-lure with the previously reported phenol-m-cresol blend elicited the highest response among the treatments tested. In a subsequent sticky-card trapping assay, traps baited with K-lure captured significantly more stable flies than the control traps. These results identify ketones as stable fly attractants and support further evaluation of K-lure in trap-based monitoring and management.

## Simple Summary

The stable fly is a blood-feeding pest of livestock worldwide. Current control methods rely mainly on sanitation, insecticides, and visually attractive traps, but these methods can be costly, labor-intensive, or less effective over time. This study aimed to identify odor chemicals that attract stable flies and could improve trap performance. We first tested materials associated with animal hosts and fly development, including chicken manure, sheep blood and blood meal, and then evaluated odor chemicals identified from these materials or selected from other animal-associated sources. We then examined their volatile chemicals and focused on a group of compounds known as ketones. Three chemicals from a group known as ketones were attractive to stable flies. A mixture of two of these ketones, referred to as K-lure, attracted significantly more flies among the ketone combination tested. In large tent enclosure trials, traps containing either K-lure caught significantly more stable flies than traps without the lure. These findings identify promising ketone-based odor cues that could be further developed as lures for stable fly monitoring and management.

## 1. Introduction

The stable fly, *Stomoxys calcitrans* (Linnaeus, 1758), is one of the most damaging blood-feeding pests of livestock worldwide [1]. Stable fly feeding causes pain, defensive behavior, reduced weight gain, decreased feed efficiency, lowered milk production and disease transmission [1,2,3]. Stable flies cause more than $2.2 billion in annual losses to U.S. cattle production and rank among the most economically important arthropod pests of cattle [4]. Stable flies also attack horses, swine, poultry, zoo animals, companion animals, and humans, extending their economic and welfare impacts well beyond cattle production systems [5,6].

Stable fly management remains difficult because adults are mobile, persistent blood feeders, and larvae develop in widely distributed organic substrates, including manure, decaying feed, bedding, and hay residues [7,8,9]. Traps are widely used to monitor adult stable-fly populations and can also contribute to population suppression. Most stable-fly traps rely primarily on visual responses to ultraviolet-reflective fiberglass, white panels, or blue–black cloth targets [10]. However, adding synthetic manure-associated volatiles to visual traps can increase stable-fly captures, demonstrating the potential value of lure-baited traps for population monitoring and suppression [11]. These studies indicate that the addition of appropriate odor cues could improve the performance of visually based stable fly traps.

Behavioral responses of stable flies are influenced by volatile cues associated with different ecological contexts. Host-associated cues, including carbon dioxide, host breath, bovine rumen volatiles, and odors associated with the skin and its microorganisms, can contribute to adult host-seeking and feeding behavior. In contrast, odors from dung and other decomposing organic substrates can provide cues associated with oviposition and larval-development sites [12,13,14,15,16,17]. Although these studies have identified behaviorally active gases, phenolic compounds, short-chain fatty acids, alcohols, terpenes, and sulfur-containing compounds, the role of ketones in stable-fly attraction remains less clearly resolved.

Ketones are organic compounds characterized by a carbonyl group bonded to two carbon atoms. Many ketones are volatile and occur in vertebrate breath, skin emissions, rumen contents, dung, and other host-associated odors, but their behavioral roles in stable flies remain poorly understood. Several ketones have been identified primarily through chemical or electrophysiological analyses [14,15,18]. Acetone has been reported to increase stable-fly activation and upwind orientation, while carvone has been associated with oviposition and increased field trap captures [18,19]. The ketone 3-octanone was identified as a major antennally active constituent of horse- and cattle-dung headspace, but it was not tested individually for behavioral attraction [14]. Similarly, 6-methyl-5-hepten-2-one has elicited electrophysiological and behavioral responses in studies involving cattle-associated flies, including *S. calcitrans*, but its value as an individual stable-fly attractant was not established [20]. These observations suggest that ketones may contribute to stable fly host- or resource-seeking behavior, but their potential as individual attractants or components of synthetic lures has received limited attention.

In this study, we first evaluated stable-fly responses to several host- and resource-associated substrates and characterized volatile compounds from attractive raw materials such as chicken manure, wet blood meal and microwaved defibrinated sheep blood using SPME-GC-MS. Because several ketones were detected in these materials and the behavioral role of this chemical class in stable flies remains comparatively underexplored, we selected ketones for systematic behavioral evaluation. Candidate ketones detected in the headspace of the attractive raw materials were screened together with ketones previously reported in published studies of other vertebrate host- and resource-associated materials. Behaviorally active ketones were subsequently evaluated in dose–response and blend assays and compared with previously reported phenol-cresol attractants. We hypothesized that selected host and resource-associated ketones would significantly attract stable flies and a blend of active ketones would yield a lure that would improve the trap catch. We therefore evaluated selected ketones individually and in blends to identify candidate lures for stable fly trapping and management.

## 2. Materials and Methods

### 2.1. Insects

A laboratory colony of stable flies (*Stomoxys calcitrans*) maintained at Apex Bait Technologies, Inc. (Santa Clara, CA, USA) was used throughout this study. The colony has been maintained under laboratory conditions for more than 10 years. Adult flies were reared at 27 ± 4 °C, approximately 50% relative humidity, and a 12 h:12 h light:dark photoperiod. Flies were provided with defibrinated sheep blood and 10% sucrose solution. Mixed-sex adults, 3–5 days old, were used in all behavioral bioassays.

### 2.2. Materials

Chicken manure was obtained from local poultry farms in California, USA. Blood meal (Burpee Natural Organic Blood Meal, 12-0-0; bovine origin) was purchased commercially. Defibrinated sheep blood was obtained from HemoStat Laboratories (Dixon, CA, USA) and stored at 4 °C until use. Candidate ketones were selected for behavioral screening based on their detection in the volatile profiles of the tested raw materials and/or previous reports of these compounds from host- and resource-associated odor sources. Synthetic compounds evaluated in this study included 2-decanone, 2-heptanone, 2-nonanone, 2-octanone, 6-methyl-5-hepten-2-one, 5-methyl-3-heptanone, 3-heptanone, 2-methyl-3-heptanone, 3-methyl-4-heptanone, 3-octanone, 6-methyl-2-heptanone, 2-butanone, acetophenone, and 1-octen-3-one. All compounds were ≥95% pure and were purchased from TCI America (Portland, OR, USA) and Sigma-Aldrich (St. Louis, MO, USA).

### 2.3. Volatile Collection and Compound Selection

Volatile compounds from chicken manure, wet blood meal, and microwaved sheep blood were collected using solid-phase microextraction (SPME). For each sample, 5 g of raw material was used, except for sheep blood, for which 5 mL was used. For wet blood meal, 5 g of blood meal was placed in a 1 oz plastic cup and mixed with 1 mL of water. Chicken manure (5 g) was weighed into a 1 oz plastic cup and used directly for volatile collection. For sheep blood, 5 mL of defibrinated sheep blood was placed in a 50 mL glass beaker (Borosil, Mumbai, India) and microwaved for approximately 5 s. Each sample was placed individually inside a 16 oz Mason jar. The jar was covered with aluminum foil and fitted with a lid containing a small opening for insertion of the SPME fiber. The sample jar was then placed inside a clean enclosed chamber (22 in wide × 14 in high) connected to a Growbright high velocity inline fan (Model GB4HV, CFM: 170, RPM: 2500, 80 Watts, 0 Amps, 120 volts, 60 Hz) fitted with an activated charcoal filter to provide purified airflow. Air was passed through the chamber at approximately 40 mL min^−1^, and the chamber remained closed during volatile collection. Headspace volatiles were collected for 30 min using a 50/30 µm DVB/CAR/PDMS SPME fiber (Supelco, Bellefonte, PA, USA). Following collection, the fiber was immediately inserted into the GC-MS injector for thermal desorption. The same SPME sampling procedure was used for all three raw materials. The fiber was conditioned between analyses, and blank runs were performed regularly to minimize background contamination and confirm the absence of carryover.

### 2.4. GC-MS Analysis

Volatile compounds were analyzed using an HP G1800A GCD system equipped with a hyperbolic metal quad analyzer (Cal-Tech Scientific, Pasadena, CA, USA) and operated using HP ChemStation software (version B.02.05). Separation was performed on a SupelcoWAX capillary column (Supelco, Bellefonte, PA, USA; 30 m × 0.25 mm i.d., 0.25 µm film thickness), with helium as the carrier gas at a constant flow rate of 1.0 mL min^−1^. The injector was maintained at 250 °C and the transfer line and MS analyzer were maintained at 280 °C. Mass spectra were acquired using electron ionization (EI) over a scan range of *m/z* 30–250. The GC oven temperature program differed slightly among the raw materials. For chicken manure, the oven was held at 50 °C for 2 min, increased at 5 °C min^−1^ to 80 °C, and then increased at 10 °C min^−1^ to 250 °C and held for 2 min. For wet blood meal and microwaved sheep blood, the oven was initially held at 50 °C for 2 min, increased at 5 °C min^−1^ to 200 °C, and then increased at 10 °C min^−1^ to 250 °C and held for 2 min. Different temperature programs were used to provide suitable chromatographic separation for the different volatile profiles of the sample types. Compounds were tentatively identified by comparison of their mass spectra with those in the NIST mass spectral library (NIST v.98) and by comparison of experimentally determined retention indices (RIs) with published values for comparable polar stationary phases. Experimental RIs were calculated according to the van den Dool and Kratz method [21] using a homologous series of *n*-alkanes (C8–C40; Sigma-Aldrich, USA) analyzed under the same chromatographic conditions as the corresponding samples. The identities of 3-octanone and 6-methyl-5-hepten-2-one were further verified using authentic standards. The identified compounds, retention times, and experimental RI values are provided in Supplementary Table S1.

### 2.5. Behavioral Bioassays

Behavioral responses of stable flies were evaluated in a screened tent measuring approximately 6 ft high × 8 ft wide × 8 ft long. A circular opening (approximately 15 inches in diameter) with a cheesecloth sleeve on the front side of the tent was used to introduce and retrieve the jar traps while minimizing disturbance and escape of flies. Glass Mason jars (16 oz; Ball, Westminster, CO, USA) wrapped with black paper covering the transparent area and fitted with custom-made paper funnels were used as traps (Supplementary Figure S1). Each funnel contained an entrance opening approximately 0.8 cm in diameter. For the raw-material assays, test materials were placed in 1 oz plastic cups inside the mason jar traps. The chicken manure assay included 5% sucrose solution (100 µL) alone or combined with 0.3 g hay, 0.3 g chicken manure, or both. Wet blood meal (2 g mixed with 4 mL distilled water) was tested against 4 mL distilled water. Room-temperature sheep blood (1 mL) was tested against 1 mL distilled water. For the comparison of blood treatments, sheep blood was microwaved for 4–5 s, allowed to return to room temperature, and then tested against refrigerated sheep blood (1 mL per treatment). All raw-material assays were conducted for 30 min. For assays involving synthetic compounds, test chemicals were prepared at the required concentrations in a proprietary solvent, with percentage concentrations expressed on a weight-to-weight basis (% *w*/*w*). The proprietary solvent alone was used as the control. For each treatment, 100 µL of the test solution or solvent control was applied using a micropipette onto a small piece of paper towel (approximately 1 × 1 inch). The treated paper towel was placed in a 1 oz plastic cup, which was then positioned inside the mason jar together with a piece of paper towel wetted with 1.0 mL water. Synthetic-compound assays were conducted for 1 h. The concentrations tested for individual candidate ketones are provided in Supplementary Table S2. Following evaluation of the individual ketones and their combinations, the two-component blend of 0.1% 3-octanone and 0.001% 1-octen-3-one prepared in the proprietary solvent was selected as the K-lure. Phenol and cresol blends were subsequently evaluated based on previous reports that phenol, *m*-cresol, and *p*-cresol are attractive to stable flies [15]. Phenol, *m*-cresol, and *p*-cresol were each tested at 0.01%. Binary blends of phenol with either *m*-cresol or *p*-cresol were evaluated alone and in combination with the K-lure to determine whether these previously reported attractants increased the behavioral response to the K-lure. Approximately 300–500 mixed-sex adult stable flies were maintained in the tent during the behavioral assays. Flies were generally 3–5 days old when initially introduced into the enclosure. The same population of flies was used across replicate assays. During the experimental period, flies were provided with 10% sucrose solution, defibrinated sheep blood, and water *ad libitum*. When the number of flies declined because of mortality or other losses, additional 3-5-day-old flies were introduced and allowed to acclimatize before subsequent testing. At the end of each assay, the entrance opening of each funnel was plugged and the traps were retrieved. Captured flies were counted individually and released back into the enclosure. Depending on the experiment, assays consisted of two-treatment choice tests, four-treatment multiple-choice tests, or six-treatment multiple-choice tests, with the corresponding control included in each assay. The chicken manure experiment was conducted with five replicate assays (*n* = 5), whereas four replicate assays were conducted for each of the other jar-trap behavioral experiments (*n* = 4). Traps were positioned approximately 3–4 ft apart, and treatment positions were rotated among replicate assays to minimize positional bias. The proportional response of stable flies to each treatment was expressed as percentage response and calculated based on the total number of responding flies within each replicate, following the approach used in insect behavioral choice assays by Revadi et al. [22] and Guidobaldi and Guerenstein [23].

$$\mathrm{Percentage}\;\mathrm{response}\;(\%)=\frac{\mathrm{number}\;\mathrm{of}\;\mathrm{flies}\;\mathrm{captured}\;\mathrm{by}\;a\;\mathrm{treatment}}{\mathrm{total}\;\mathrm{number}\;\mathrm{of}\;\mathrm{flies}\;\mathrm{captured}\;\mathrm{across}\;\mathrm{all}\;\mathrm{treatments}\;\mathrm{within}\;\mathrm{the}\;\mathrm{replicate}}\times 100$$

### 2.6. Sticky-Card Enclosure Assays

Following the behavioral bioassays, the selected two-component K-lure was further evaluated using a sticky-card trapping assay in the same screened tent described above. White sticky cards (6 × 8 in; Qualirey, Shenzhen, China) were mounted on black A4 cardstock (Astrobrights, Alpharetta, GA, USA), leaving an approximately 1.5 in black border around the sticky surface (Supplementary Figure S2). Two traps were positioned on opposite sides of the tent. The K-lure consisted of 0.1% (*w*/*w*) 3-octanone and 0.001% (*w*/*w*) 1-octen-3-one prepared in a proprietary solvent. The K-lure and proprietary solvent control were placed separately in 1.5 mL microcentrifuge tubes (Kashi Scientific, Portland, OR, USA), which served as lure dispensers. Each dispenser contained 1 mL of either K-lure or proprietary solvent. Approximately 500 mixed-sex adult stable flies were maintained in the enclosure and were generally 3–5 days old when initially introduced. Flies were allowed to acclimatize for 3 days before testing. During the acclimatization period and throughout the experiment, flies were provided with 10% sucrose solution, refrigerated defibrinated sheep blood, and water *ad libitum*. The K-lure and control traps were left in place for 24 h, after which the traps were removed and the number of stable flies captured on each sticky card was counted manually. Percentage response was calculated as the number of flies captured on each treatment trap relative to the total number of flies captured on both traps within each replicate. Four replicate trapping assays (*n* = 4) were conducted. Treatment positions were alternated between replicate assays to minimize positional bias. Because flies were removed through trap capture or lost through natural mortality, approximately 100–150 additional 3–5-day-old flies were introduced between replicate assays, as needed, to maintain a relatively consistent fly population within the enclosure. Newly introduced flies were allowed to acclimatize for 24 h before subsequent testing.

### 2.7. Statistical Analysis

Percentage response data from the behavioral assays, including the sticky-card trapping assay, were analyzed using the Mann–Whitney test or the Kruskal–Wallis test, as appropriate. The Kruskal–Wallis test was followed by Dunn’s multiple comparisons post hoc test. Differences were considered statistically significant at *p* < 0.05. All statistical analyses were performed using GraphPad Prism version 9.0.0 (GraphPad Software, San Diego, CA, USA).

## 3. Results

### 3.1. Behavioral Responses of Stable Flies to Host- and Resource-Associated Materials

Stable fly responses differed significantly among treatments containing chicken manure (Figure 1A; Kruskal–Wallis: χ^2^ = 16.30; *df* = 3; *p* = 0.001, *n* = 5). The combination of 5% sucrose and chicken manure elicited the highest response (mean ± s.e.m; 54.83 ± 2.24%) and was significantly more attractive than the 5% sucrose control (5.10 ± 0.41%). Wet blood meal was also significantly more attractive to stable flies than control (Figure 1B; Mann–Whitney; *p* = 0.02, *n* = 4), eliciting a mean response of 80.58 ± 4.78% compared with 19.42 ± 4.78% for the control. Similarly, sheep blood at room temperature (RT) elicited a significantly greater response than the control (Figure 1C; Mann–Whitney; *p* = 0.02, *n* = 4), with mean responses of 91.11 ± 4.23% and 8.89 ± 4.23%, respectively. Microwaved sheep blood also elicited a significantly greater response than refrigerated sheep blood (Figure 1D; Mann–Whitney; *p* = 0.02, *n* = 4), with mean responses of 97.58 ± 1.51% and 2.42 ± 1.51%, respectively. Based on these behavioral responses, chicken manure, wet blood meal, and microwaved sheep blood were selected for volatile analysis.

### 3.2. Volatile Profiles of Selected Attractive Materials

SPME-GC-MS analysis of chicken manure, wet blood meal, and microwaved sheep blood revealed chemically diverse volatile profiles containing several ketones and other volatile compounds (Figure 2). Candidate ketones identified from these materials, together with selected literature-guided compounds and structural analogues, were subsequently screened for stable fly attraction (Supplementary Table S2).

### 3.3. Screening and Dose-Dependent Responses of Candidate Ketones

Fourteen candidate ketone volatiles were selected for screening based on GC–MS analysis of headspace volatiles collected from attractive raw materials and compounds reported in the published literature. Because the compounds were evaluated during different stages of the screening program, they were tested over compound-specific dose or concentration ranges (Supplementary Table S2). Of the candidate ketones evaluated, 3-octanone, 1-octen-3-one, and 6-methyl-5-hepten-2-one elicited significant behavioural response and were selected for further dose–response evaluation. Stable fly attraction to 3-octanone differed significantly among concentrations in both dose response assays. In the first assay, 0.10% 3-octanone produced the highest mean response (Figure 3A; 47.59 ± 5.84%; Kruskal–Wallis: χ^2^ = 12.85, *df* = 3, *p* < 0.0001). In the second assay, attraction was again highest at 0.10% (Figure 3B; 39.24 ± 4.61%; χ^2^ = 13.06, *df* = 3, *p* < 0.0001), whereas the response declined at 0.30%. Attraction to 1-octen-3-one also differed significantly among concentrations (Figure 3C; Kruskal–Wallis: χ^2^ = 12.75; *df* = 3; *p* < 0.0001). Attraction was highest at 0.001% and declined at higher concentrations. Testing of lower concentrations showed the highest response among the concentrations tested occurred at 0.001% as the optimal concentration (Figure 3D; Kruskal–Wallis: χ^2^ = 11.50; *df* = 3; *p* = 0.0008). Stable fly attraction to 6-methyl-5-hepten-2-one differed significantly among concentrations (Figure 3E; Kruskal–Wallis: χ^2^ = 10.58; *df* = 3; *p* = 0.0027). Attraction was highest at 0.01% and declined at 0.10%. Based on these assays, 0.10% 3-octanone, 0.001% 1-octen-3-one, and 0.01% 6-methyl-5-hepten-2-one were selected for subsequent comparison and blend assays.

### 3.4. Comparative Behavioral Activity of Individual Ketone Attractants and Evaluation of the K-Lure

The three active ketones were compared at their respective selected concentrations. Stable fly attraction differed significantly among treatments (Figure 4A; Kruskal–Wallis: χ^2^ = 10.04; *df* = 3; *p* = 0.004). The 0.10% 3-octanone treatment produced the highest response, with 43.04 ± 6.96% attraction compared with 7.03 ± 2.92% for the control.

Binary and ternary combinations of the three active ketones differed significantly in stable fly attraction (Figure 4B; Kruskal–Wallis: χ^2^ = 19.09; *df* = 5; *p* = 0.001). The combination of 0.10% 3-octanone (K2) and 0.001% 1-octen-3-one (K3) produced the highest response among the ketone combinations and was designated the K-lure.

The effectiveness of the K-lure was further evaluated using sticky trap assay. Sticky traps baited with the K-lure showed a significantly greater percentage response than the control traps (Figure 4C; Mann–Whitney U; *p* = 0.02). The K-lure-baited traps captured 66.25 ± 2.75% of the flies compared with 33.75 ± 2.75% for the control traps. These results demonstrate that the attractiveness of the K-lure observed in the behavioral assays was also retained when deployed with sticky traps.

### 3.5. Behavioral Response of Stable Flies to K-Lure Combined with Phenol and Cresol Blends

Stable fly response differed significantly among the K-lure and phenol-cresol treatments (Figure 5; Kruskal–Wallis: χ^2^ = 21.79; *df* = 5; *p* = 0.0006). The K-lure combined with phenol and *m*-cresol produced the highest overall response (34.15 ± 2.91%) and was significantly greater than the control.

## 4. Discussion

Host- and substrate-associated odors provided a useful source of candidate stable fly attractants. In this study, chicken manure, microwaved defibrinated sheep blood, and wet blood meal were attractive in behavioral assays and were therefore used as sources for headspace volatile analysis. GC-MS analysis of these attractive materials, together with compounds reported in the published literature [24,25], provided a targeted group of ketones for behavioral screening. Previous stable fly studies have similarly used attractive host- or substrate-associated materials to identify volatile compounds for subsequent electrophysiological or behavioral testing [13,15,26,27].

The results identified 3-octanone, 1-octen-3-one, and 6-methyl-5-hepten-2-one as behaviorally active compounds and showed that stable fly responses varied among the concentrations tested, with reduced responses at higher concentrations for some compounds. These results are consistent with earlier semiochemical attractant studies of stable flies. The compound 6-methyl-5-hepten-2-one previously increased stable fly upwind flight at specific concentrations in laboratory assays, while 3-octanone was identified as a predominant chemostimulant in attractive horse and cattle dung odors used by gravid stable flies [14,20]. The present study extends these observations by demonstrating behavioral attraction to these ketones when tested individually.

Dose–response assays showed that each active ketone had a distinct optimal concentration. The highest attraction was observed at 0.10% for 3-octanone, 0.001% for 1-octen-3-one, and 0.01% for 6-methyl-5-hepten-2-one. Attraction to 1-octen-3-one and 6-methyl-5-hepten-2-one declined when the concentration was increased above the optimum. A similar concentration-dependent response has been reported for other stable fly semiochemicals; phenol and cresols showed greater attraction at lower doses, and synthetic host-associated odor blends have also shown reduced activity when presented at higher concentrations [15,16]. Together, these studies show that increasing the concentration of a behaviorally active volatile does not necessarily increase stable fly attraction and may reduce the response at higher concentrations, highlighting the importance of concentration and volatile release rate when developing controlled-release formulations for trap use.

Among the three active ketones, 3-octanone produced the strongest individual response at its optimal concentration. Combining 3-octanone with 1-octen-3-one produced the highest response among the tested ketone combinations and was designated the K-lure. The addition of phenol and m-cresol to the K-lure produced the highest overall response among the formulations evaluated. Previous stable fly studies have also shown that combining host-associated odorants can alter attraction relative to individual compounds. Tangtrakulwanich et al. [15] found that binary blends of phenol with m-cresol or p-cresol were particularly attractive, and field traps baited with these binary blends also captured more stable flies. Recent work with synthetic cattle-breath volatiles further showed that combining attractive cues does not necessarily produce an additive response. Kovacs et al. [17] found that synthetic cattle-breath volatiles attracted stable flies, but adding CO_2_ and CH_4_ did not increase landings above the synthetic volatile blend alone.

Importantly, the activity of the K-lure was maintained when it was evaluated with sticky traps under large tent trapping assays. K-lure-baited traps captured approximately twice as many stable flies as control traps, providing additional evidence that the behavioral activity observed in the multiple-choice assays can translate to an applied trapping system. However, these experiments were conducted under enclosed large tent trapping conditions, where fly movement and environmental variation were more restricted than under open-field conditions. Field evaluation is therefore needed to determine whether the increased trap capture is maintained under livestock production conditions.

The present screening process narrowed the candidate volatiles from attractive raw materials to three active ketones and subsequently to defined concentrations and combinations for lure development. The concentration-dependent responses observed here also indicate that formulation and volatile release will be important during field development of the lure. Previous field studies with cattle-associated flies have shown that attractants identified under laboratory conditions may produce different responses in the field when release rates are not optimized [27]. Therefore, the next step will evaluate the K-lure under field conditions using controlled-release formulations.

The identification of behaviorally active ketones and the development of the K-lure provide a foundation for developing stable fly-specific attractant formulations. The K-lure has potential to be incorporated into trap-based systems or bait platforms to improve stable fly detection, capture, and localized management. The feasibility of this application is supported by previous field studies in which m-cresol was incorporated directly into attractant-impregnated adhesive traps and m-cresol-baited traps were subsequently used as the “pull” component of a stable fly push–pull management system on pasture cattle [28,29]. Field trials will determine the most effective formulation, dispenser, and deployment strategy under livestock production conditions.

## 5. Conclusions

This study identified 3-octanone, 1-octen-3-one, and 6-methyl-5-hepten-2-one as attractive ketones for stable flies and showed that their activity was concentration-dependent. A binary blend of 3-octanone and 1-octen-3-one, designated K-lure, produced the strongest response among ketone combinations. These results support further development of K-lure as a stable fly attractant for trap-based monitoring and management under livestock production conditions.

## Figures and Tables

**Figure 1:**
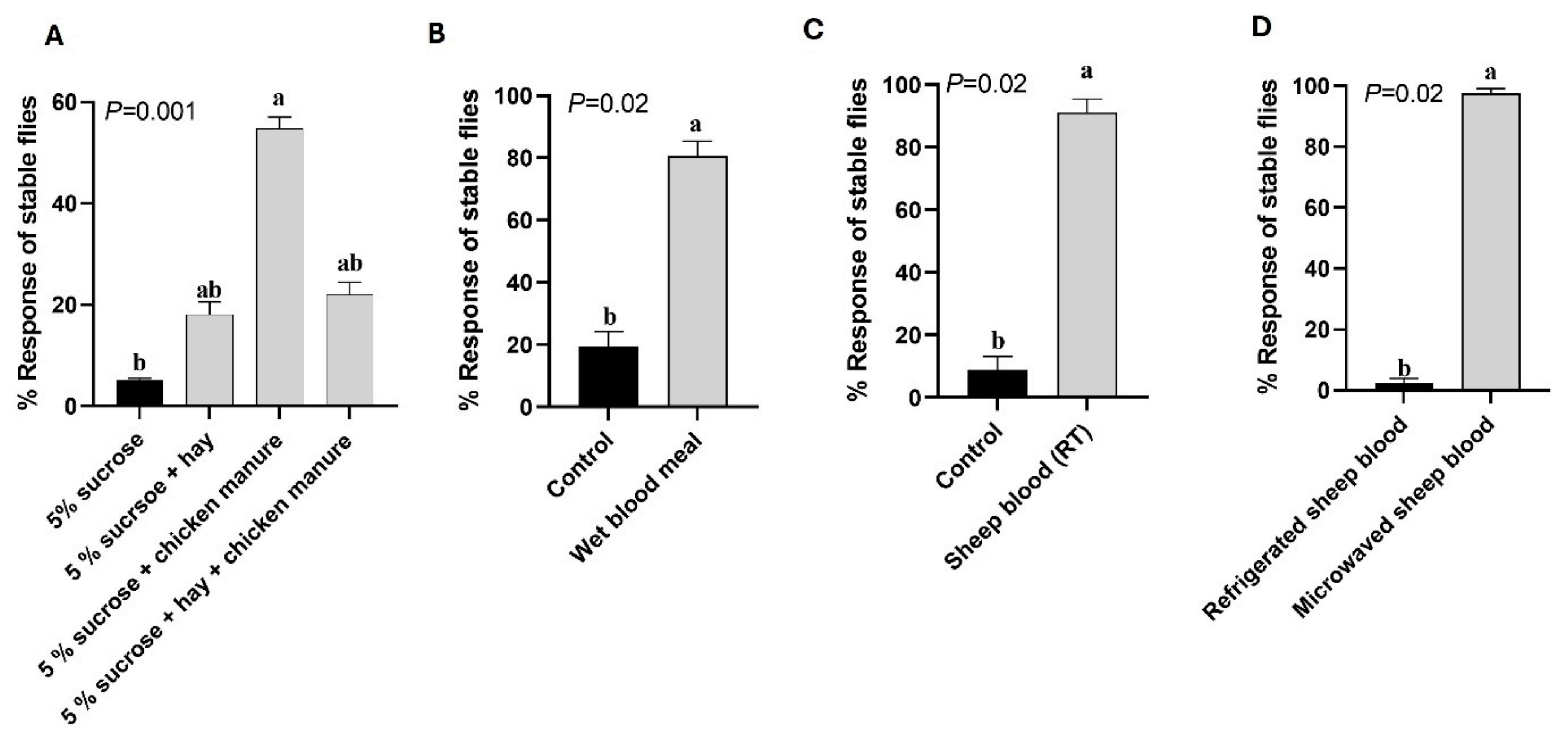
Behavioral responses of stable flies to selected raw materials. (**A**) Four choice assay evaluating 5% sucrose solution alone or combined with hay, chicken manure, or both (*n* = 5). (**B**) Two-treatment choice assay evaluating wet blood meal compared with distilled water as the control (*n* = 4). (**C**) Two-treatment choice assay evaluating room-temperature (RT) sheep blood compared with distilled water as the control (*n* = 4). (**D**) Two-treatment choice assay evaluating microwaved sheep blood compared with refrigerated sheep blood (*n* = 4). Bars represent mean percentage response ± SEM. Different letters above the bars indicate significant differences among treatments (*p* < 0.05).

**Figure 2:**
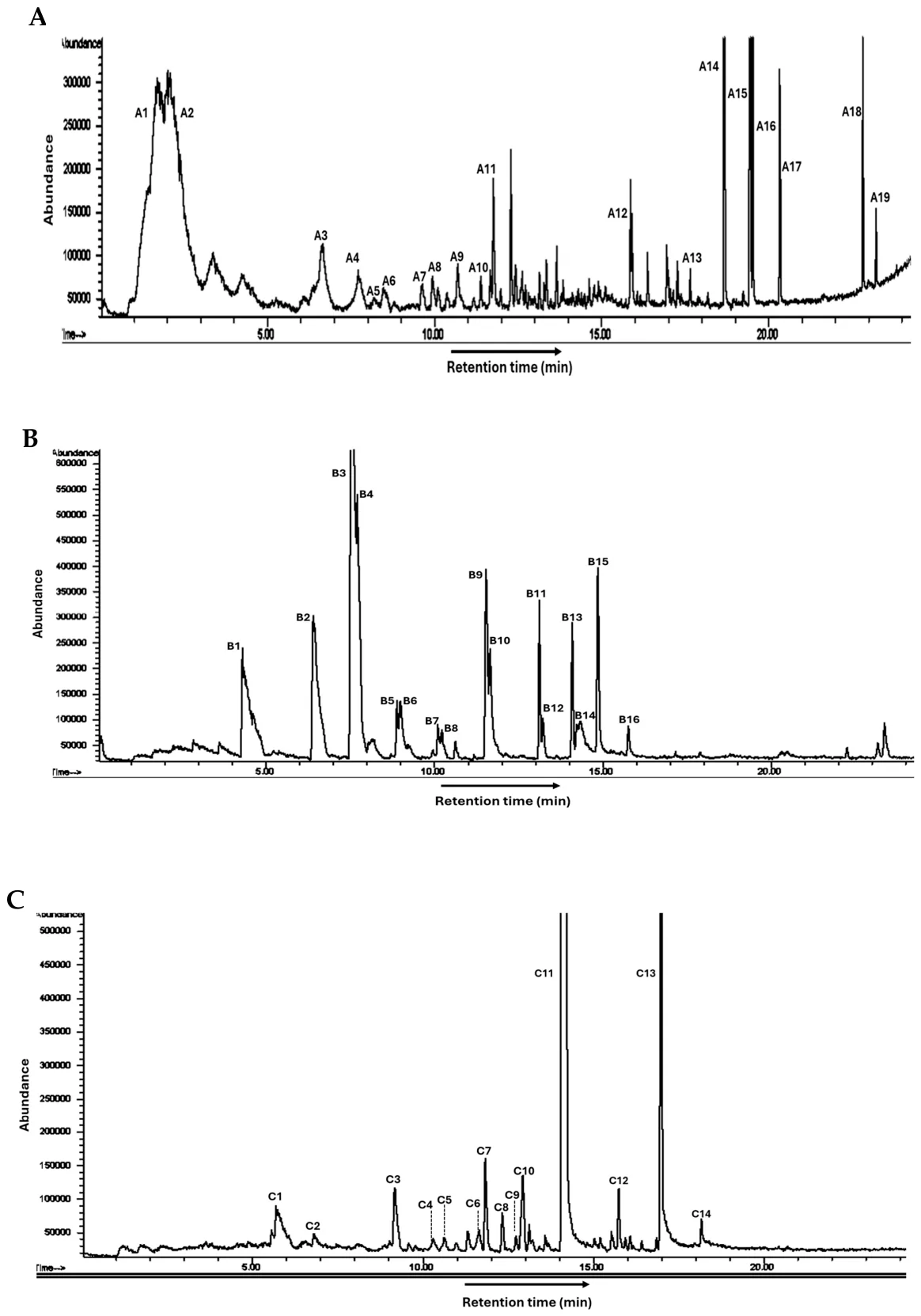
Representative SPME-GC-MS chromatograms of volatile compounds collected from (**A**) chicken manure, (**B**) wet blood meal, and (**C**) microwaved sheep blood using solid-phase microextraction (SPME). Labeled peaks correspond to compounds tentatively identified by mass spectral library matching and retention-index comparison and are listed in Supplementary Table S1. Selected compounds were further verified using authentic standards. The y-axis represents GC–MS detector signal intensity (abundance) in arbitrary units (a.u.).

**Figure 3:**
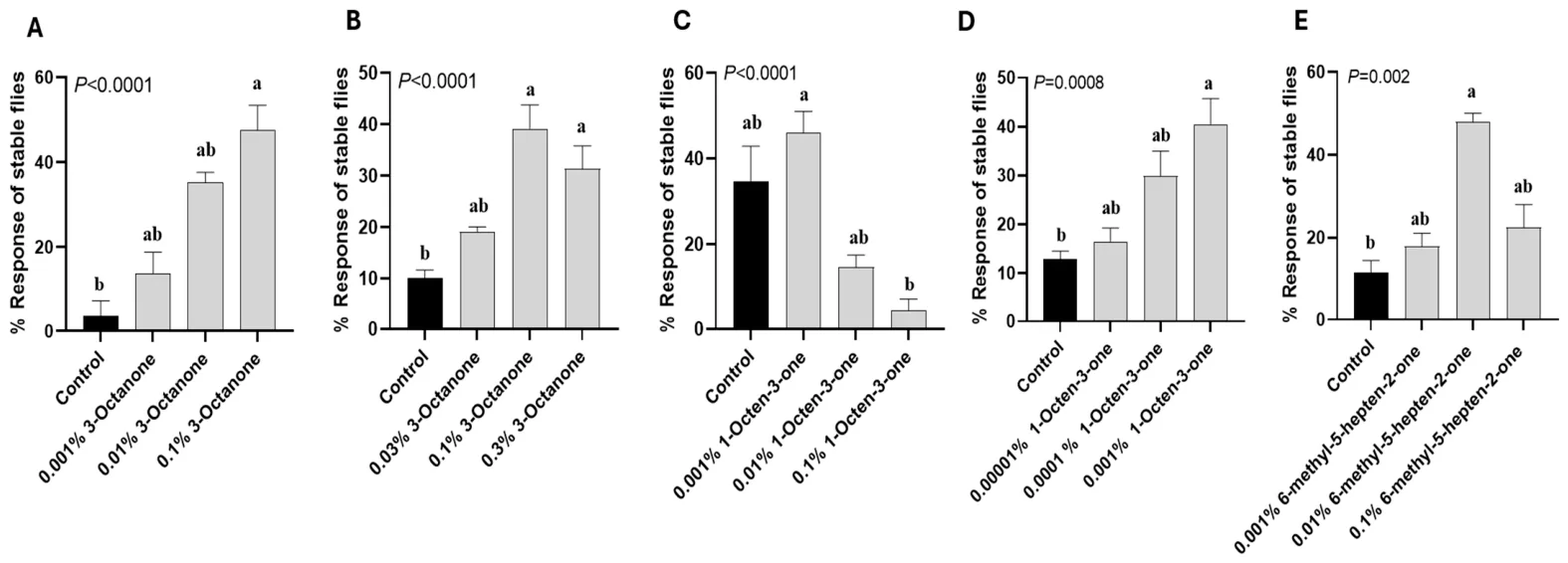
Dose-dependent behavioral responses to the three active ketones. (**A**) 3-octanone at 0.001–0.1%; (**B**) subsequent 3-octanone assay at 0.03–0.3%; (**C**) 1-octen-3-one at 0.001–0.1%; (**D**) 1-octen-3-one at 0.00001–0.001%; and (**E**) 6-methyl-5-hepten-2-one at 0.001–0.1%. Each assay included the proprietary solvent control (*n* = 4). Bars represent mean percentage response ± SEM. Different letters above the bars indicate significant differences among treatments based on Dunn’s multiple-comparison test (*p* < 0.05).

**Figure 4:**
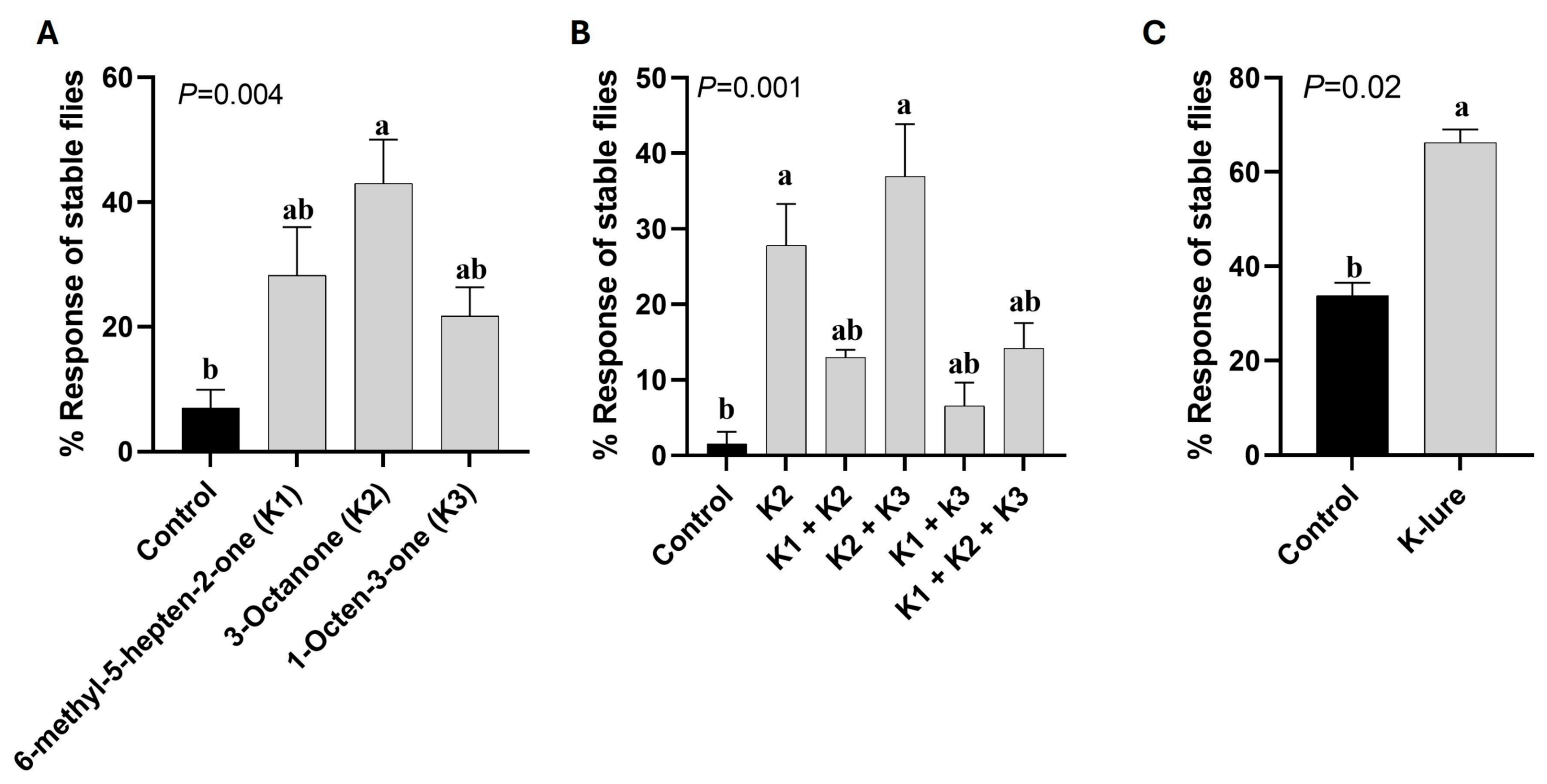
Behavioral responses of stable flies to selected ketones and the K-lure. (**A**) Percentage response to the three individual ketones at their selected concentrations: K1, 0.01% 6-methyl-5-hepten-2-one; K2, 0.1% 3-octanone; and K3, 0.001% 1-octen-3-one (*n* = 4). (**B**) Percentage response to binary and ternary combinations of the three ketones (*n* = 4). The K-lure consisted of K2 + K3 (0.1% 3-octanone + 0.001% 1-octen-3-one). (**C**) Percentage response of stable flies captured on sticky-card traps baited with the K-lure or proprietary solvent control during the 24 h trapping period (*n* = 4). Bars represent mean ± SEM. Different letters above the bars indicate significant differences among treatments (*p* < 0.05).

**Figure 5:**
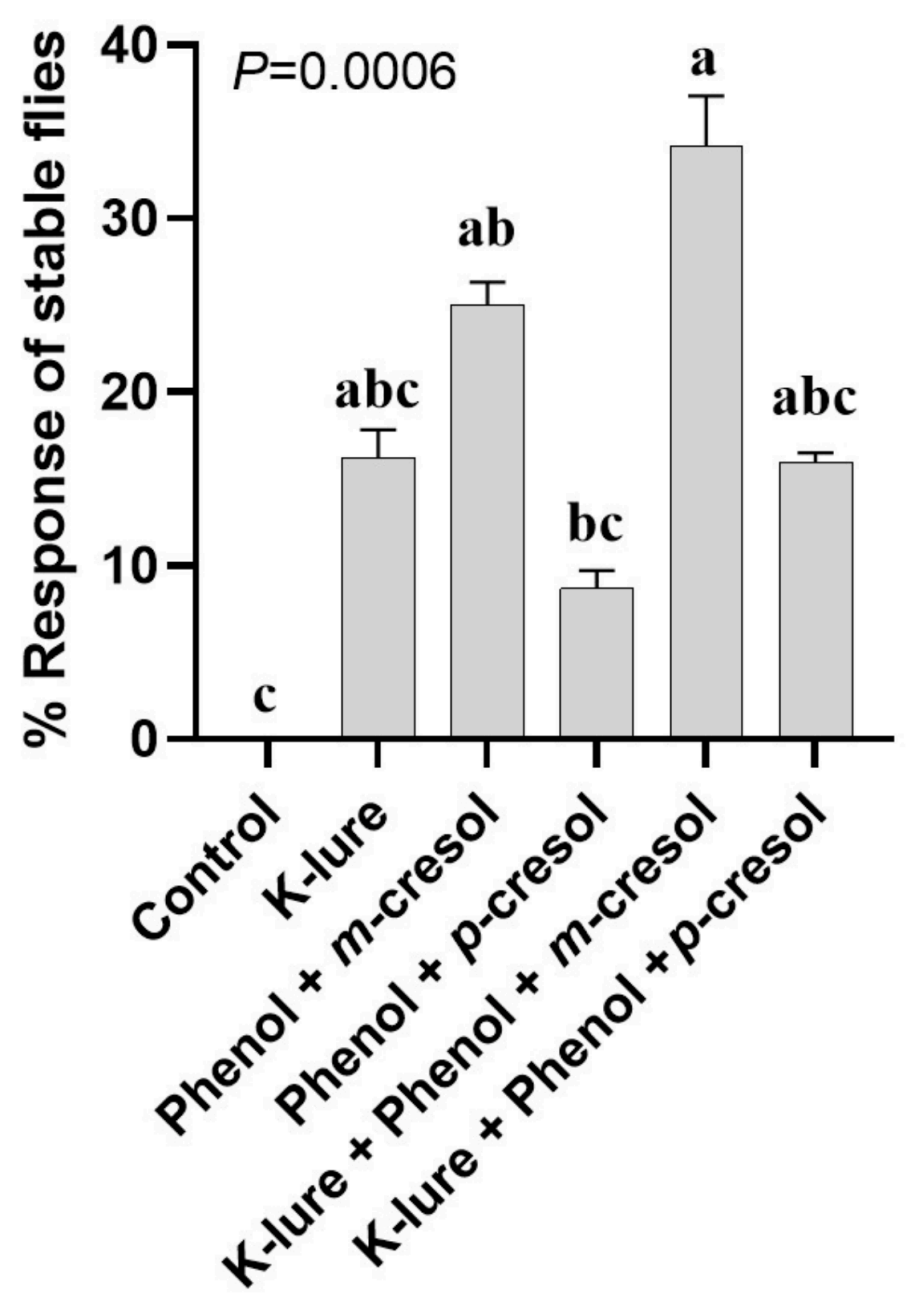
Behavioral responses of stable flies to the K-lure and combinations with phenol and cresol blends. Phenol, *m*-cresol, and *p*-cresol were each tested at 0.01%. The K-lure consisted of 0.1% 3-octanone and 0.001% 1-octen-3-one. Bars represent mean percentage response ± SEM (*n* = 4). Different letters above the bars indicate significant differences among treatments (*p* < 0.05).

## Data Availability

The data supporting the findings of this study are provided in the article and Supplementary data S1.

## Supplementary Materials

The following supporting information can be downloaded at: https://www.mdpi.com/article/10.3390/insects17101071/s1, Figure S1: Mason jar traps used for the stable fly behavioral bioassays; Figure S2: (A) Large tent used for stable fly behavioral bioassays. (B) Sticky-card traps used for evaluation of the K-lure; Table S1: GC-MS identification of volatile compounds detected in chicken manure, wet blood meal, and microwaved sheep blood; Table S2: Candidate ketones selected from SPME-GC-MS analyses of attractive raw materials, structural analogues and published host-associated volatiles, together with the dose ranges tested and behavioral responses of stable flies (*Stomoxys calcitrans*); Supplementary Data S1: Raw behavioral bioassay data.
