# Supplementary material for: Identification and Behavioral Evaluation of Ketone Attractants for the Stable Fly, Stomoxys calcitrans
Source: Insects. 2026 Oct 16;17(10):1071. doi: 10.3390/insects17101071 (PMC13705007; doi:10.3390/insects17101071)

Supplementary Figure S1. Mason jar traps used for the stable fly behavioral bioassays.

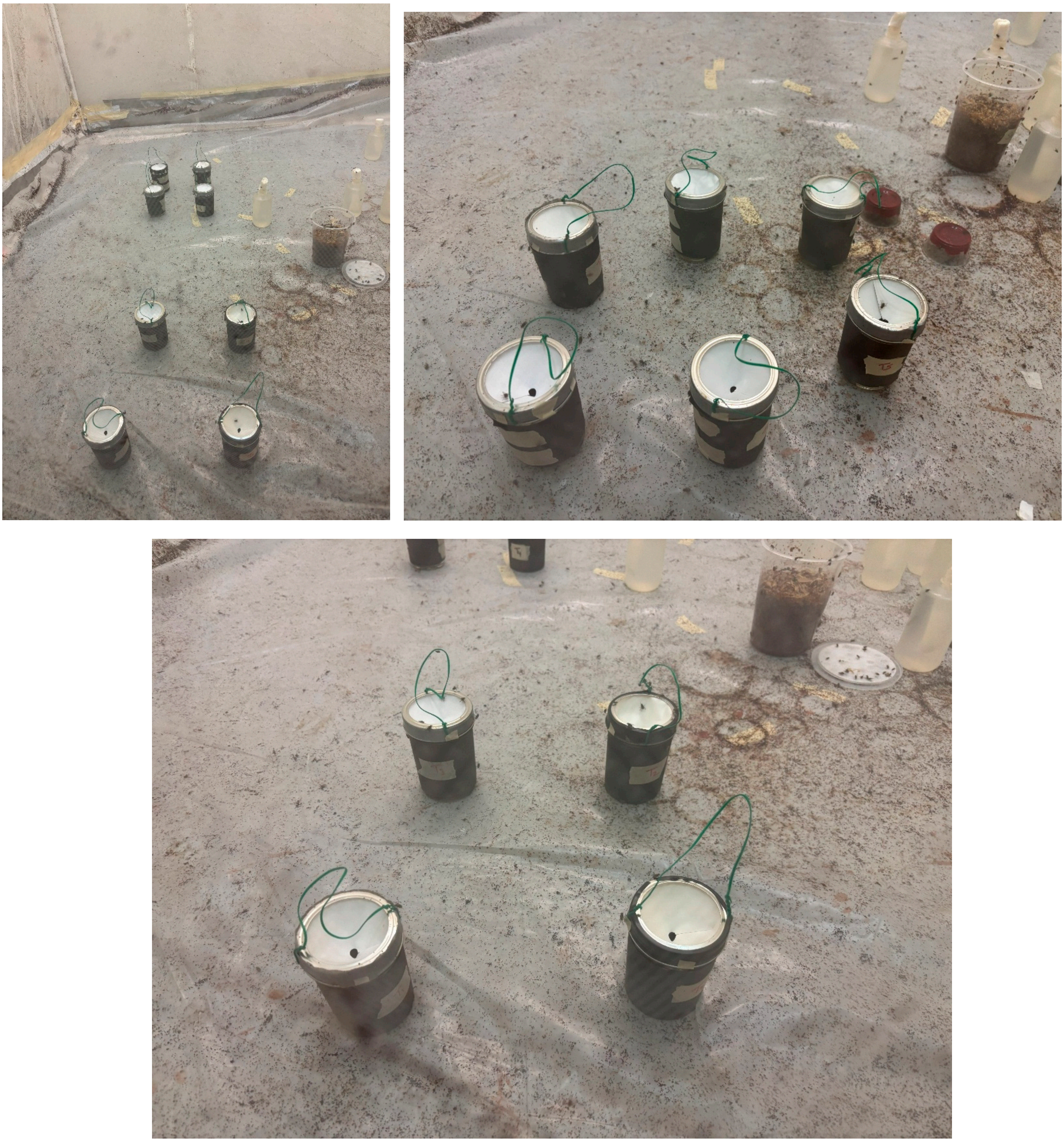

Supplementary Figure S2. (A) Large tent used for stable fly behavioral bioassays. (B) Sticky-card traps used for evaluation of the K-lure.

(A)

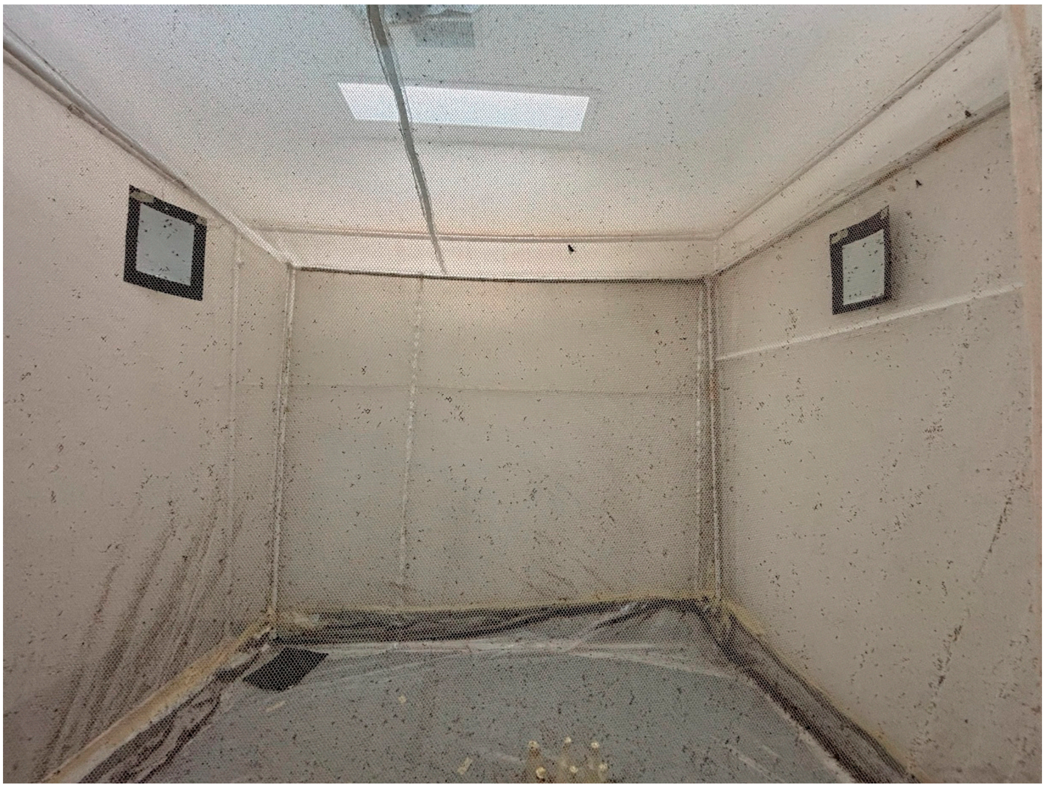

(B)

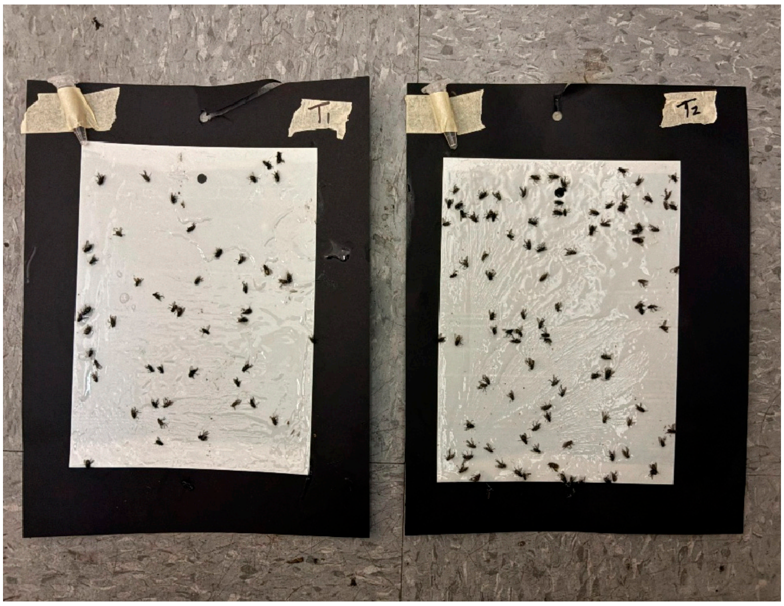

Supplement: Supplementary file 1 [file insects-17-01071-s001.zip › Supplementary Figures S1 and S2.pdf]
