# Supplementary material for: Identification and Behavioral Evaluation of Ketone Attractants for the Stable Fly, Stomoxys calcitrans
Source: Insects. 2026 Oct 16;17(10):1071. doi: 10.3390/insects17101071 (PMC13705007; doi:10.3390/insects17101071)
Supplement: Supplementary file 1 [file insects-17-01071-s001.zip › Supplymentary Table S1.pdf]

Supplementary Table S1. GC-MS identification of volatile compounds detected in chicken manure, wet blood meal, and microwaved sheep blood.

Sample	Peak	Compound	Retention time (min)	Experimental RI
**Chicken manure**	A1	Acetone	1.697	ND
	A2	2-Butanone	2.078	ND
	A3	3-Methyl-1-butanol	6.657	1199
	A4	3-Octanone*	7.715	1244
	A5	6-Methyl-2-heptanone	8.180	1264
	A6	2-Octanone	8.459	1276
	A7	6-Methyl-5-hepten-2-one *	9.633	1331
	A8	1-Hexanol	9.938	1346
	A9	3-Octanol	10.690	1384
	A10	1-Octen-3-ol	11.665	1440
	A11	3-Butenyl isothiocyanate	11.756	1446
	A12	Aniline	15.906	1742
	A13	2-Phenylethyl alcohol	17.650	1897
	A14	Phenol	18.667	1994
	A15	*p*-Cresol	19.435	2070
	A16	*m*-Cresol	19.518	2078
	A17	4-Ethylphenol	20.328	2161
	A18	Indole	22.817	2433

	A19	Skatole	23.218	2479
**Wet blood meal**	B1	Hexanal	4.298	1080
	B2	2-Heptanone	6.406	1187
	B3	2-Pentylfuran	7.531	1235
	B4	6-Methyl-2-heptanone	7.705	1242
	B5	2-Octanone	8.879	1290
	B6	Octanal	8.995	1295
	B7	5-Methyl-3-hepten-2-one	10.103	1339
	B8	6-Methyl-5-hepten-2-one	10.221	1343
	B9	2-Nonanone	11.532	1394
	B10	Nonanal	11.650	1399
	B11	1-Octen-3-ol	13.113	1456
	B12	1-Heptanol	13.220	1460
	B13	2-Ethyl-1-hexanol	14.093	1494
	B14	2-Decanone	14.306	1502
	B15	Benzaldehyde	14.849	1524
	B16	1-Octanol	15.758	1561
**Microwaved sheep blood**	C1	3-Heptanone	5.693	1153
	C2	3-Methyl-2-heptanone	6.793	1205
	C3	3-Heptanol	9.158	1302
	C4	3-Methyl-3-hepten-2-one	10.281	1345

C5	1-Hexanol	10.635	1359
C6	3-Octanol	11.296	1385
C7	Cyclohexanal	11.828	1406
C8	1-Ethylcyclohexanol	12.323	1425
C9	2-Ethyl-4-methyl-1-pentanol	12.725	1441
C10	1-Octen-3-ol	13.114	1456
C11	2-Ethyl-1-hexanol	14.200	1498
C12	1-Octanol	15.747	1560
C13	2-Hexyloxyethanol	16.974	1610
C14	1-Nonanol	18.167	1660

Note: Compounds were tentatively identified by comparison of their mass spectra with the NIST mass spectral library and experimentally determined retention indices (RIs) with published RI values for comparable polar stationary phases. Experimental RIs were calculated using a homologous series of *n*-alkanes (C8-C40) analyzed under the same chromatographic conditions as the corresponding samples. **ND**, not determined because the compound eluted before the first reliably resolved *n*-alkane reference.

***** Identification was further verified using an authentic standard.
