# Supplementary material for: Identification and Behavioral Evaluation of Ketone Attractants for the Stable Fly, Stomoxys calcitrans
Source: Insects. 2026 Oct 16;17(10):1071. doi: 10.3390/insects17101071 (PMC13705007; doi:10.3390/insects17101071)
Supplement: Supplementary file 1 [file insects-17-01071-s001.zip › Supplymentary Table S2.pdf]

Supplementary Table S2. Candidate ketones selected from SPME-GC-MS analyses of attractive raw materials, structural analogues and published host-associated volatiles, together with the dose ranges tested and behavioral responses of stable flies (*Stomoxys calcitrans*).

No.	Compound	Basis for selection	Detected in / Selection source *	Dose range tested†	Stable fly response
1	2-Decanone	SPME- GC-MS + literature	Wet blood meal	1-100 µg	No significant attraction
2	2-Heptanone	SPME- GC-MS + literature	Wet blood meal	1-100 µg	No significant attraction
3	2-Nonanone	SPME- GC-MS + literature	Wet blood meal	5-500 µg	No significant attraction
4	2-Octanone	SPME- GC-MS + literature	Wet blood meal; Chicken manure	5-500 µg	No significant attraction
5	**6-Methyl-5-hepten-2-one**	SPME- GC-MS + literature	Wet blood meal; Chicken manure	0.001-0.1%	**Attractive (highest response at 0.01%)**
6	5-Methyl-3-heptanone	Structural analogue	Sheep blood analogue compounds	0.1-10 µg	No significant attraction
7	3-Heptanone	SPME-GC-MS	Microwaved sheep blood	Multiple dose ranges (5-500 µg; 1-100 µg; 0.01-1%)	No significant attraction
8	2-Methyl-3-heptanone	Structural analogue	Sheep blood ketone analogue	0.1-10 µg	No significant attraction

9	3-Methyl-4-heptanone	Structural analogue	Sheep blood ketone analogue	0.1-10 µg	No significant attraction
10	**3-Octanone**	SPME- GC-MS + literature	Chicken manure	0.001-0.3%	**Attractive (highest response at 0.1%)**
11	6-Methyl-2-heptanone	SPME-GC-MS	Wet blood meal; Chicken manure	1-100 µg	No significant attraction
12	2-Butanone	SPME-GC-MS	Chicken manure	5 µg	No significant attraction
13	Acetophenone	Literature-guided candidate	Published host-associated volatile	0.001-0.1%	No significant attraction
14	**1-Octen-3-one**	Literature-guided candidate; metallic/blood-like odorant associated with skin lipid oxidation	Not detected in the raw materials analyzed by HS-SPME–GC–MS	0.00001-0.1%	**Attractive (highest response at 0.001%)**

* Detected in / Selection source indicates either the raw material(s) in which a compound was detected by SPME-GC-MS in the present study or the source used for candidate selection.

† Candidate ketones were screened over the dose ranges available during the study. Dose ranges are reported in their original experimental units (mass doses or percentage solutions) to reflect the screening protocol used for each compound.

Structural analogue = Compound selected as a structural analogue of ketones associated with sheep blood.
